# Baseline systemic inflammatory indices and clinicopathological features to predict the outcome of acute tubulointerstitial nephritis: A single-center retrospective study

**DOI:** 10.1007/s00508-024-02417-2

**Published:** 2024-08-27

**Authors:** Ahmet Burak Dirim, Nazrin Namazova, Merve Guzel Dirim, Ozgur Akin Oto, Ayse Serra Artan, Ozge Hurdogan, Yasemin Ozluk, Halil Yazici

**Affiliations:** 1 Division of Nephrology, Department of Internal Medicine, Faculty of Medicine, Istanbul University, 34104 Istanbul, Turkey; 2 Department of Internal Medicine, Faculty of Medicine, Istanbul University, Istanbul, Turkey; 3 Department of Pathology, Faculty of Medicine, Istanbul University, Istanbul, Turkey

**Keywords:** Drugs, Treatment, Steroids, Prognosis, End-stage kidney disease

## Abstract

**Background:**

Acute tubulointerstitial nephritis (AIN) is an immune-mediated disorder that can cause acute kidney injury (AKI). We aimed to investigate the characteristics of patients with AIN and predictive factors for treatment response.

**Material and methods:**

In this study, thirty-one patients diagnosed with AIN on kidney biopsy between 2006 and 2021 were included. Baseline clinical, histopathological, and laboratory findings, including complete blood count (CBC), creatinine, erythrocyte sedimentation rate, C‑reactive protein, C3, C4, systemic immune inflammation index (SII), neutrophil-to-lymphocyte ratio (NLR), platelet-to-lymphocyte ratio (PLR), and urinalysis were evaluated. Treatment response, mortality, and creatinine levels at the time of last follow-up were also noted.

**Results:**

The median age was 46 years and 80.6% were female. Median baseline creatinine and proteinuria levels were 4.1 mg/dL and 0.84 gram/day. The median follow-up period was 14 months and 93.5% received immunosuppressives. End-stage kidney disease (ESKD) developed in five patients (16.1%). Renal recovery (creatinine < 1.4 mg/dL) was observed in 17 patients (54.8%). Higher degrees of interstitial fibrosis, tubular atrophy, granuloma formation, global glomerulosclerosis, and higher baseline hemoglobin levels, in addition to a longer interval between first symptom to initiation of immunosuppressives were associated with renal nonrecovery, statistically. Also, patients who progressed to ESKD had higher baseline hemoglobin (*p* = 0.033) and lymphocyte (*p* = 0.044) and lower PLR levels (*p* = 0.016), as well as higher degrees of global glomerulosclerosis (*p* = 0.014), interstitial fibrosis (*p* = 0.042), and tubular atrophy (*p* = 0.030).

**Conclusion:**

Treatment response rates are low for AIN, which may lead to ESKD. Besides chronicity in histopathology specimens, higher baseline hemoglobin levels and lower platelet-to-lymphocyte ratio might be prognostic. Further studies should be conducted on new markers for AIN.

**Supplementary Information:**

The online version of this article (10.1007/s00508-024-02417-2) contains supplementary material, which is available to authorized users.

## Introduction

Acute tubulointerstitial nephritis (AIN) is characterized by acute tubulointerstitial inflammatory infiltration involving the tubules and interstitium of the kidney parenchyma, which can cause acute kidney injury (AKI). Because AIN is treatable, it should not be overlooked [1]. In the past years, the most common cause of AIN has been infections; however, drugs are currently the leading cause, and drug-induced AIN accounts for approximately 70–90% of all AIN cases. Additionally, AIN can occur due to systemic diseases, such as sarcoidosis, Sjogren’s syndrome, systemic lupus erythematosus (SLE), immunoglobulin G4-related disease, and tubulointerstitial nephritis and uveitis (TINU) syndrome. In recent years, immune checkpoint inhibitor-related AIN and COVID-19 and COVID-19 vaccine-related AIN cases have been reported. Clinical and laboratory findings of allergic reactions, including rashes, fever, and eosinophilia, can be observed in addition to abnormal urinary sediment findings and mild proteinuria (< 1 g/day)*. *Most patients with AIN experience AKI, and early diagnosis and treatment improve prognosis [1–3]. The first treatment approach in drug-induced AIN is discontinuation of the drug in question. In addition, the treatment of underlying infections or systemic diseases in AINs related to these situations is sine qua non. Corticosteroids can be administered in drug-related AIN that does not improve after cessation of the drug or in systemic disorder-related AIN, such as sarcoidosis and Sjogren’s disease [1, 2, 4]. Hematologic parameters, including the systemic immune inflammation index (SII), neutrophil-to-lymphocyte ratio (NLR), and platelet-to-lymphocyte ratio (PLR) were shown to be possible predictive factors in acute kidney injury [5]; however, the prognostic role of these indices in AIN has not been previously studied. In this retrospective single-center study the aim was to investigate the clinical, histopathological, and laboratory characteristics, including the SII, NLR, and PLR, and predictive factors for treatment response in patients diagnosed with AIN between 2006–2021.

## Material and methods

### Study population and evaluation of the patients

Patients diagnosed with AIN via renal biopsy between 2006 and 2021 were included in this retrospective study. These patients had no history of chronic kidney disease and underwent renal biopsy because of unexplained renal dysfunction. The baseline clinical, laboratory, and histopathological findings in addition to clinical data, including edema, blood pressure, and AIN etiology, were recorded. Prior history of hypertension, diabetes, and additional clinical findings were also noted. Baseline laboratory findings (at the time of renal biopsy), including complete blood count (CBC), systemic immune inflammation index (SII = neutrophil × platelet/lymphocyte), serum neutrophil-to-lymphocyte ratio (NLR), platelet-to-lymphocyte ratio (PLR), serum creatinine, estimated glomerular filtration rate (eGFR) according to the CKD-EPI equation [6], electrolytes, serum lipid profile, C‑reactive protein (CRP), autoimmune serology, serum C3 and C4 levels, serum albumin, serum total protein, erythrocyte sedimentation rate (ESR), urinalysis, venous blood gas, and spot urine protein-to-creatinine ratio (uPCR) or 24‑h urine proteinuria (g/day) were evaluated. Histopathological findings, including percentage of global and segmental sclerotic glomeruli; degree of tubulointerstitial inflammatory infiltration; grade of tubular atrophy; interstitial fibrosis; and the presence of granuloma formation, were recorded. In addition, the treatment, clinical status, and mortality rates during follow-up were evaluated. Recovery of kidney function was defined as a serum creatinine level < 1.4 mg/dL at the last control. Patients were divided into two groups according to the last creatinine level (patients with serum creatinine < 1.4 mg/dL at the time of last follow-up were classified as responders and those with serum creatinine ≥ 1.4 mg/dL as non-responders). End-stage kidney disease (ESKD) was defined as the initiation of chronic renal replacement therapy.

### Histopathological assessment

Kidney biopsy specimens were evaluated using light microscopy, immunofluorescence, and electron microscopy. Histological and immunofluorescence stains were prepared using 3–4 µm-thick sections. For staining, 0.4–0.6 cm fresh tissue was frozen in liquid nitrogen, cut sections were stained for immunofluorescence antibodies and graded on a semiquantitative scale from 0 to 3. The remaining tissues were fixed in Hollande’s fixative, embedded in paraffin, and processed routinely for light microscopic evaluation using various stains, such as hematoxylin and eosin, periodic acid-Schiff, methenamine silver-periodic acid, Masson’s trichrome, and Congo red. Interstitial fibrosis and tubular atrophy were evaluated using a semi-quantitative scale from 0 to 3. Interstitial infiltration of lymphocyte, eosinophil, and plasma cells and the presence of granuloma were graded on a scale of 0 to 3. The specimens were fixed in glutaraldehyde and embedded in Epon blocks for electron microscopy in selected cases.

### Statistical analysis

In the descriptive statistics, the mean, standard deviation, median, minimum, maximum, and frequency values were used. The distribution of variables was measured using the Kolmogorov-Smirnov test. The Mann-Whitney U test was used to analyze quantitatively independent data. The analysis of qualitatively independent data was performed using the χ^2^-test. Fisher’s exact test was performed when the χ^2^-test conditions were not met. SPSS® (version 26.0; IBM®, Armonk, NY, USA), was used for data analysis. Statistical significance was set at *p* < 0.05. Receiver operating characteristic (ROC) curve analysis was performed for statistically significant variables to predict the development of ESKD.

## Results

### Demographic/clinical and follow-up data of patients

The demographic and clinical data of the study population are shown in Table 1, 80.6% were female, and the median age was 46 years. Edema was observed in 19.4% at admission. Median time from the first symptom (e.g., fatigue, nausea, edema or asymptomatic renal dysfunction in the laboratory results) to renal biopsy was 21 days (min:3–max:90 days) (The patient, who underwent biopsy 90 days after the first symptom, had drug-related AIN. Prolonged time for admission was linked to intermittent use of NSAIDs. This patient had a good response to corticosteroids, and serum creatinine level was 0.77 mg/dL at the last follow-up). The mean systolic and diastolic blood pressures were 132.9 and 82.1 mmHg, respectively, and 7 patients (22.6%) required hemodialysis at first admission. Prior history of hypertension and diabetes was present in 19.4% and 6.5%, respectively. A family history of renal disease was present in one non-responder patient with Sjögren’s disease-related AIN.

**Table 1:** Demographics, baseline laboratory and clinical data of the patients

Baseline parameters (at time of renal biopsy)	
Median age (years, min-max)	46 (17–71)
Gender, female/male (%)	25/6 (80.6%/19.4%)
Mean systolic blood pressure, mm Hg (±SD)	132.9 ± 17.9
Mean diastolic blood pressure, mm Hg (±SD)	82.1 ± 12.1
Prior hypertension, *n* (%)	6 (19.4%)
Prior diabetes mellitus, *n* (%)	2 (6.5%)
Time from first symptom to biopsy (days), median (min-max)	21 (3–90)
Edema (%)	6 (19.4%)
Mean hemoglobin, g/dL (±SD)	10.4 ± 2
Median WBC, per mm^3^ (min-max)	7740 (3900–29,300)
Median neutrophil, per mm^3^ (min-max)	5800 (3100–24,700)
Median Plt, per µl (min-max)	260,000 (75,000–645,000)
Median Lym, per mm^3^ (min-max)	1300 (400–3300)
Median Eos, per mm^3^ (min-max)	185 (0–700)
Median serum creatinine, mg/dL (min-max)	4.1 (1.03–11.2)
Median eGFR (CKD-EPI), ml/min/1.73 m^2^ (min-max)	13 (3.7–70)
Median BUN, mg/dL (min-max)	32.7 (10–103)
Median urea, mg/dL (min-max)	72.5 (25–210)
Median uric acid, mg/dL (min-max)	5.6 (2–14.3)
Mean serum sodium (mmol/L) (±SD)	138.6 ± 3.7
Mean serum potassium (mmol/L) (±SD)	4.3 ± 0.8
Mean serum albumin, g/dL (±SD)	4.0 ± 0.6
Mean serum calcium, mg/dL (±SD)	9.1 ± 0.8
Median serum phosphorus, mg/dL (min-max)	3.9 (2.3–8.8)
Median ESR, mm/hr (min-max)	69 (5–151)
Median CRP, mg/L (min-max)	28 (1–220)
Mean serum total protein, g/dL	7.1 ± 1.1
Median serum C3, mg/dL (min-max)	128 (73–201)
Median serum C4, mg/dL (min-max)	32 (20–79)
Median serum LDH, U/L (min-max)	191 (114–512)
Median LDL, mg/dL (min-max)	103 (53–517)
Median TG, mg/dL (min-max)	139.5 (65–611)
Median total cholesterol, mg/dL (min-max)	180.5 (115–652)
Median AST, U/L (min-max)	19 (8–93)
Median ALT, U/L (min-max)	15 (7–43)
Median proteinuria, g/g or g/day (min-max)	0.84 (0–8)
Median urine erythrocyte, per HPF	3 (0–99)
Median urine leukocyte, per HPF	3 (0–84)
Median follow-up time, months (min-max)	14 (1–168)

*ALT* alanine aminotransferase, *AST* aspartate aminotransferase, *BUN* blood urine nitrogen, *CRP* C reactive protein, *eGFR* estimated glomerular filtration rate, *Eos* eosinophil, *ESR* erythrocyte sedimentation rate, *HPF* high power field, *LDH* lactate dehydrogenase, *LDL* low-density lipoprotein, *Lym* lymphocyte count, *Plt* platelet, *SD* standard deviation, *TG* triglyceride, *WBC* white blood cell count

The etiologies of AIN are listed in Table 2. The most common cause of AIN in our cohort was unknown (35.5%). Other etiologies of AIN included antibiotics (22.6%), non-steroidal anti-inflammatory drugs (NSAIDs) (22.6%), sarcoidosis (12.9%), and Sjögren’s disease (6.5%) in our study group. All patients with unknown etiology were screened for autoimmune systemic disorders and multiple myeloma with serological tests, such as ANA, Anti-ds DNA, ANCA, serum C3, C4, and protein electrophoresis. No specific etiology of AIN was identified in these patients. On the other hand, 4 among 11 patients had regular drug use, including antihypertensive treatment, oral antidiabetics, and statins. Of these four patients two developed ESKD during follow-up. Hence, drug-related AIN could not be excluded in these four patients. Additionally, the cause of AIN in three patients who progressed to ESKD could not be fully determined according to the data in the patient files. Another responder patient with an unknown etiology had anti-cardiolipin IgG and IgM antibodies; however, other serological and clinical findings were not compatible with autoimmune connective tissue disorders. Similarly, one non-responder patient (developed ESKD during follow-up) had ANA positivity. Nevertheless, the serological and clinical findings were not compatible with autoimmune diseases. In addition, migraine and osteoarthritis diagnoses were present in the remaining two patients but these patients had no history of nonsteroidal anti-inflammatory drug (NSAID) exposure. Therefore, the etiology could not be classified.

**Table 2:** Etiology and renal biopsy characteristics of AIN patients

		*n* (%)
Etiology	Unknown	11 (35.4%)
	NSAID	7 (22.6%)
	Antibiotics	7 (22.6%)
	Sjögren’s disease	2 (6.5%)
	Sarcoidosis	4 (12.9%)
Tubulitis	Not significant	5 (16.1%)
	Mild	23 (74.2%)
	Moderate	1 (3.2%)
	Severe	2 (6.5%)
Lymphocytic inf	Not significant	1 (3.2%)
	Mild	23 (74.2%)
	Moderate	5 (16.1%)
	Severe	2 (6.5%)
Eosinophilic inf	Not significant	17 (54.8%)
	Mild	8 (25.8%)
	Moderate	2 (6.5%)
	Severe	4 (12.9%)
Plasma cell inf	Not significant	22 (71%)
	Mild	1 (3.2%)
	Moderate	1 (3.2%)
	Severe	7 (22.6%)
Granuloma formation	None	24 (77.4%)
	Mild/microgranuloma*	4 (12.9%)
	Moderate (focal)	2 (6.5%)
	Extensive	1 (3.2%)
Interstitial fibrosis	None	24 (77.4%)
	Mild	6 (19.4%)
	Moderate	1 (3.2%)
	Severe	0 (0%)
Tubular atrophy	None	17 (54.8%)
	Mild	13 (41.9%)
	Moderate	1 (3.2%)
	Severe	0 (0%)

*Inf* infiltration, *NSAID* Non-steroidal anti-inflammatory drugs

* The presence of just one granuloma or microgranuloma formation in entire biopsy sample

Among 31 patients 93.5% received immunosuppressive treatment after AIN diagnosis (Table 2). Immunosuppressive treatment, including pulse steroids, were started after the biopsy diagnosis in all except two patients. One of these patients had antibiotic (gentamycin)-related AIN and required hemodialysis at admission. Owing to the active infective state, immunosuppressants could not be implemented. After the cessation of gentamycin treatment, renal function was ameliorated (creatinine level was 0.6 mg/dL at the last control). Another female patient had NSAID-related AIN. After cessation of NSAID, renal function began to improve until biopsy results were obtained. Creatinine level decreased to 1.4 mg/dL from 4.1 mg/dL in 1 month; however, the patient was lost to follow-up and was classified as a non-responder. In our cohort, the most common initial steroid regimen was prednisolone (1 mg/kg/day). After 1–2 weeks, the corticosteroid dosage was reduced and discontinued after 4–6 weeks of treatment in responders. Azathioprine or mycophenolate mofetil was administered to steroid-refractory or steroid-dependent cases. All patients in our cohort had at least 1 month of follow-up. The median follow-up period was 14 months. Of these patients 54.8% were responders, 5 patients (16.1%) progressed to ESKD during the follow-up period and 3 patients died during follow-up. All deceased patients were in the non-responder group.

### Laboratory data of the patients

Baseline laboratory data (at time of renal biopsy), including CBC, serum creatinine, estimated glomerular filtration rate (eGFR) according to the CKD-EPI equation, electrolytes, serum lipid profile, C‑reactive protein (CRP), autoimmune serology, serum C3 and C4 levels, serum albumin, serum total protein, erythrocyte sedimentation rate (ESR), urinalysis, and spot urine protein-to-creatinine ratio (UPCR) or 24‑h proteinuria (g/day) are shown in Table 1. The median creatinine level of the patients was 4.1 mg/dL. The only patient with a serum creatinine 1.03 mg/dL had prior Sjogren’s disease, and was under hydroxychloroquine treatment. A renal biopsy was performed due to acute-onset nephrogenic diabetes insipidus. The biopsy was consistent with AIN. Therefore, steroid and mycophenolic acid were administered. After that, diabetes insipidus resolved, and the patient was included in the responder group.

Venous blood gas analysis results were present in 22 patients (11 responders and 11 non-responders) at time of renal biopsy. Metabolic acidosis was present in 9 of the 22 (40.9%) patients. All 9 patients (7 of 11 responders vs. 2 of 11 non-responder patients, *p* = 0.08, Fischer’s exact test) had a normal anion gap (Na-(Cl+HCO3) < 12 mEq/L) metabolic acidosis (actual HCO3 concentration < 22 mEq/L) that could be related to renal tubular dysfunction as a consequence of AIN.

### Histopathological data of the patients

Tubulitis, tubulointerstitial eosinophils, lymphocytes, and plasma cell infiltration degrees as well as the grade of tubular atrophy and interstitial fibrosis in the biopsy specimens, are shown in Table 2. The median glomeruli count was 12 (5–35). The median percentage of global and segmental sclerotic glomeruli were 2.86% (0–83.33%) and 0% (0–9%), respectively. The patient with 83.3% of global sclerosis had inadequate biopsy sample. One of the two cores was lack of cortex and consisted of medulla, and the other core was a scanty subcapsular sample with 83.3% of global sclerotic glomeruli.

### Laboratory, clinical, treatment and histopathological data of responder and non-responder patients

The laboratory, clinical, and histopathological data of the responders are shown in Tables 3 and 4. Median time from first symptom to immunosuppressive treatments was longer in non-responder patients than responders (45 days, min: 13/max: 80 days vs. 18.5 days, min: 3/max: 96 days; *p* *=* 0.028). Responder patients had statistically lower hemoglobin levels compared to non-responders (median 9.75 vs. 11.8 g/dL, *p* = 0.029). Other baseline laboratory results, treatment regimens, and etiologies of AIN were similar in both groups (Table 3). Non-responders had higher degrees of granuloma formation (*p* = 0.012), tubular atrophy (*p* = 0.024), interstitial fibrosis (*p* = 0.047), and a higher percentage of global sclerotic glomeruli (*p* = 0.013) compared to the responders (Table 4).

**Table 3:** Clinical/laboratory and treatment data of responder (Last serum creatinine < 1.4 mg/dL) and non-responder groups

Baseline (at time of renal biopsy) parameters and follow-up data	Non-responder (*n* = 14)	Responder (*n* = 17)	*p* value
Age, years, median (min-max)	44 (21–61)	44.5 (17–71)	0.874 ^m^
Gender, female/male	10/4	15/2	0.370 _F_
Prior history of hypertension, *n* (%)	3 (21.4)	3 (17.6)	1.000 _F_
Prior history of diabetes mellitus, *n* (%)	2 (14.2)	0 (0)	0.196 _F_
Family history of kidney disease, *n* (%)	1 (7.1)	0 (0)	1.000 _F_
Time from first symptom to biopsy (day), median (min-max)	30 (5–67)	14 (3–90)	0.059 ^m^
Serum creatinine, median (min-max)	3.8 (2.4–9.1)	4.32 (1.03–11.2)	0.677 ^m^
Serum uric acid, median (min-max)	5.9 (3.5–14.3)	4.1 (2–7.8)	0.176 ^m^
eGFR (CKD-EPI), median (min-max)	16.9 (4.7–32)	11.29 (3.69–70)	0.525 ^m^
Serum albumin, mean ± SD	3.9 ± 0.8	4.0 ± 0.4	0.573 ^m^
Serum CRP, median (min-max)	25 (1–220)	32 (1–180)	0.508 ^m^
ESR, median (min-max)	66 (25–117)	79.5 (5–151)	0.605 ^m^
Serum LDH, median (min-max)	182 (114–512)	168.5 (129–335)	0.482 ^m^
Serum C3, median (min-max)	119 (73–162)	136 (90–201)	0.614 ^m^
Serum C4, median (min-max)	25 (20–59)	32 (26–79)	0.173 ^m^
**Hemoglobin, median (min-max)**	**11.8 (6.9–13.8)**	**9.75 (7.2–11.7)**	**0.029 **^**m**^
Lymphocyte, median (min-max)	1400 (400–3300)	1180 (500–1700)	0.140 ^m^
WBC, median (min-max)	9300 (3900–29,300)	7640 (5150–11,700)	0.461 ^m^
Neutrophil, median (min-max)	6500 (3100–24,700)	5650 (3270–9900)	0.665 ^m^
Platelet, median (min-max)	280,000 (75,000–645,000)	29,1500 (148,600–507,000)	0.733 ^m^
Eosinophil, median (min-max)	200 (0–700)	135 (0–300)	0.151 ^m^
SII, median (min-max)	1400 (513.6–5689.8)	1635 (495.7–4563)	0.603 ^m^
NLR, median (min-max)	4.88 (2.3–17.6)	4.97 (2.6–12.6)	0.603 ^m^
PLR, median (min-max)	194.3 (124.2–1123.5)	302.26 (151.6–460.9)	0.225 ^m^
Proteinuria, median (min-max)	0.84 (0–8)	0.84 (0–3)	0.766 ^m^
Urine leucocyte count, median (per HPF)	3	3.5	0.842 ^m^
Urine erythrocyte count, median (per HPF)	1	3.5	0.059 ^m^
Follow-up time (months), median (min-max), median (min-max)	14 (1–52)	10.5 (2–168)	0.402 ^m^
**Time from first symptom to immunosuppressive treatment (days) (min-max)**	**45 (13–80)**	**18.5 (3–96)**	**0.028 **^**m**^
Mortality rate, *n* (%)	3 (21.4)	0 (0)	0.081 _F_
*Treatment, n (%)*			
Steroids including pulse	13 (92.9)	16 (94.1)	1.000 _F_
AZA/MMF	3 (21.4)	0 (0)	0.304 _F_
Pulse steroids	4 (28.6)	3 (17.6)	0.671 _F_
Hemodialysis at admission	4 (28.6)	3 (17.6)	0.671 _F_
*Etiology of AIN, n (%)*	Unknown, 7 (50)	Unknown, 4 (23.5)	0.153 _F_
	NSAID, 2 (14.3)	NSAID, 5 (29.4)	0.412 _F_
	Antibiotic, 1 (7.1)	Antibiotic, 6 (35.3)	0.094 _F_
	Sarcoidosis, 3 (21.4)	Sarcoidosis, 1 (5.9)	0.304 _F_
	Sjögren, 1 (7.1)	Sjögren, 1 (5.9)	1.000 _F_

Bold characters indicate statistically significant values

*AIN* acute interstitial nephritis, *AZA* azathioprine, *Bx* biopsy, *CRP* c reactive protein, *eGFR* estimated glomerular filtration rate, *ESR* erythrocyte sedimentation rate, *F* Fisher’s exact test, *HPF* high power field, *LDH* lactate dehydrogenase, *Lym* lymphocyte, *m* Mann-Whitney u, *MMF* mycophenolate mofetil, *NLR* neutrophil-to-lymphocyte ratio, *NSAID* non-steroidal anti-inflammatory drugs, *PLR* platelet-to-lymphocyte ratio, *SD* standard deviation, *SII* systemic immune inflammation index, *WBC* white blood cell count

**Table 4:** Histopathological data of responder (serum creatinine < 1.4 mg/dL) and non-responder groups

Parameters	Non-responder (*n* = 14)	Responder (*n* = 17)	*p* value
*Tubulitis, n (%)*			
Not significant	3 (21.4)	2 (11.8)	0.467 _X_^2^
Mild	8 (57.1)	15 (88.2)	
Moderate	1 (7.1)	0 (0)	
Severe	2 (14.3)	0 (0)	
*Lymphocytic infiltration, n (%)*			
Not significant	1 (7.1)	0 (0)	0.452 _X_^2^
Mild	9 (64.3)	14 (82.3)	
Moderate	3 (21.4)	2 (11.8)	
Severe	1 (7.1)	1 (5.9)	
*Eosinophilic infiltration, n (%)*			
Not significant	8 (57.1)	9 (52.9)	0.815 _X_^2^
Mild	3 (21.4)	5 (29.4)	
Moderate	1 (7.1)	1 (5.9)	
Severe	2 (14.3)	2 (11.8)	
*Plasmacytic infiltration, n (%)*			
Not significant	8 (57.1)	14 (82.3)	0.124 _X_^2^
Mild	1 (7.1)	0 (0)	
Moderate	1 (7.1)	0 (0)	
Severe	4 (28.6)	3 (17.7)	
*Granuloma formation, n (%)*			
No granuloma	7 (50)	17 (100)	**0.012 **_**X**_^**2**^
Mild/microgranuloma	4 (28.6)	0 (0)	
Moderate	2 (14.3)	0 (0)	
Extensive	1 (7.1)	0 (0)	
*Tubular atrophy, n (%)*			
None	4 (28.6)	13 (76.5)	**0.024 **_**X**_^**2**^
Mild	9 (64.3)	4 (23.5)	
Moderate	1 (7.1)	0 (0)	
Severe	0 (0)	0 (0)	
*Interstitial fibrosis, n (%)*			
None	8 (57.1)	16 (94.1)	**0.047 **_**X**_^**2**^
Mild	5 (35.7)	1 (5.9)	
Moderate	1 (7.1)	0 (0)	
Severe	0 (0)	0 (0)	
*Global sclerotic glomeruli (%), median (min-max)*	11.81 (0–83.3)	0 (0–50)	**0.013 **^**m**^
Segmental sclerotic glomeruli (%), median (min-max)	0 (0–9)	0 (0–0)	0.270 ^m^

Bold characters indicate statistically significant values

*m* Mann-Whitney U test, *x*^*2*^ χ2-test

### Laboratory, clinical, treatment, and histopathological data of ESKD and non-ESKD patients

The laboratory, clinical, and histopathological data of ESKD patients are shown in supplemental Tables 1 and 2. The ESKD patients had statistically higher hemoglobin (median 12.05 vs. 9.9 g/dL, *p* = 0.033) and lymphocyte (median 2300 vs. 1260 per mm^3^, *p* = 0.044) levels than non-responders. Moreover, ESKD patients had lower PLR (median 154.38 vs. 230.36, *p* = 0.016) compared to non-ESKD patients. Other baseline laboratory results and treatment regimens were similar in both groups; however, the etiology of AIN was unknown in all ESKD patients (100% in ESKD vs. 23.1% in non-ESKD patients, *p* = 0.003) (Supplemental Table 1). Patients with ESKD had higher degrees of tubular atrophy (*p* = 0.030), interstitial fibrosis (*p* = 0.042), and higher percentage of global sclerotic glomeruli (*p* = 0.014) compared to patients without ESKD (Supplemental Table 2).

### ROC curve analysis for baseline PLR to predict end stage kidney disease

**Fig. 1:**
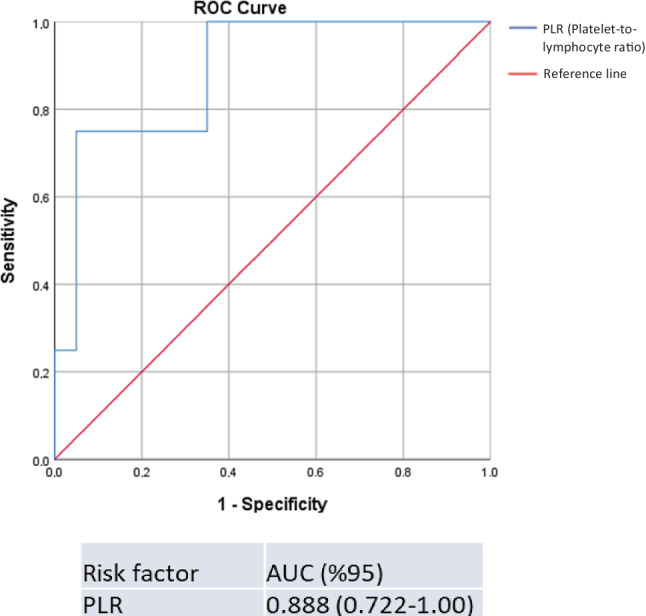
ROC curve analysis (area under curve) for baseline PLR to predict ESKD development. PLR platelet-to-lymphocyte ratio

Statistically, baseline PLR was the most significant laboratory variable in terms of ESKD development (*p* = 0.016; Supplemental Table 1). The receiver operating characteristic (ROC) curve analysis was performed to measure the area under the curve. The area under the ROC curve for PLR was 88.8% with a standard error of 5% (Fig. 1).

## Discussion

This retrospective single-center study investigated the clinical, histopathological, and laboratory findings, as well as treatment regimens and outcomes of patients with biopsy-proven AIN. The most common etiology of AIN was drug use (45.2% NSAID and antibiotics) in our cohort. Higher baseline hemoglobin levels, percentage of globally sclerotic glomeruli, interstitial fibrosis, tubular atrophy, and granuloma formation on renal biopsy, in addition to a longer interval between first symptom to initiation of immunosuppressives, were associated with poor renal outcomes. Additionally, patients who developed ESKD had higher baseline lymphocyte counts and lower PLR levels than non-ESKD patients.

Anemia can be observed in AIN [2]. Defective erythropoietin (EPO) synthesis in peritubular interstitial cells due to tubulointerstitial infiltration may be responsible for this phenomenon. Interestingly, higher baseline hemoglobin levels, regardless of creatinine or eGFR levels, were associated with poor renal outcomes in our cohort. Although baseline EPO levels and hematinic parameters, including ferritin, transferrin saturation, folic acid, and vitamin B12 levels, were unknown and none of these patients were under EPO treatment; our finding could be related to paradoxical severe interstitial infiltration. Secondary polycythemia due to EPO secretion could occur in the setting of severe leukemia and lymphoma infiltration, as seen in the cases reported by Osumi et al. [7] and Bhat et al. [8]. These patients had mild renal dysfunction, bilateral renomegaly, and increased EPO levels [7, 8]. Therefore, it could be speculated that hemoglobin and EPO levels might have a U-shaped pattern in interstitial nephritis according to the degree of interstitial inflammatory cell infiltration. In addition, the percentage of female patients was higher in the responder group compared to non-responders (88.2% vs. 71.4%), which could have contributed to the lower hemoglobin levels in the responders; however, one of the most probable explanations for this observation might be statistical error due to limited number of patients in the study group.

The remaining laboratory results, including the baseline CBC and systemic inflammation indices, were similar between responders and non-responders; however, patients who developed ESKD had higher baseline lymphocyte counts and lower PLR than non-ESKD patients. Higher levels of peripheral lymphocytes may represent more severe tubulointerstitial lymphocyte infiltration. On the other hand, no statistical difference in the degree of lymphocyte infiltration in ESKD and non-ESKD patients were observed, which might be related to the limited number of patients. To the best of our knowledge, the role of PLR in AIN prognosis has not yet been studied. Higher preoperative and early postoperative PLR levels were previously shown to be associated with AKI [9]. Another study showed that PLR has a U-shaped pattern for mortality in critically ill patients with AKI [10]. The lower PLR might be a novel marker for ESKD development in patients with AIN, according to the results of our study. Nevertheless, the limited number of patients with ESKD in our cohort might have caused this finding to be inaccurate. Further studies are required to elucidate these.

It is not surprising that patients with severe tubular atrophy, global glomerular sclerosis, and granuloma formation in renal biopsies show poor treatment responses. Similar findings have previously been reported in literature [11–13]. Histopathological findings generally represent renal damage and the possibility of the treatment response in nephrology practice. In contrast, biomarkers could be crucial for patients with AIN in situations restraining a renal biopsy, such as coagulopathy. A recent study showed that the urinary CXCL9-to-creatinine ratio could be a specific marker for AIN diagnosis [14]. Therefore, an AIN diagnosis can be established without renal biopsy. Therefore, new prognostic serum or urine markers may be beneficial for AIN. A previous study showed that urine IL‑9 and TNF‑α might be prognostic markers in AIN [11, 15]. We evaluated baseline laboratory results, including inflammation indices, as potential prognostic markers in our study.

The optimal treatment modality for patients with AIN is still not well known. In drug-related AIN, the cessation of the accused drug may be therapeutic. An older retrospective observational study showed no additional benefit of corticosteroids in AIN compared to supportive treatment [16]. In contrast, refractory cases may require immunosuppressive treatment, mainly corticosteroids. The optimal steroid dose for AIN treatment has not yet been determined. A previous study showed no difference between the efficacy of high-dose pulse therapy and oral steroids in drug-induced AIN [17]. In addition, longer intervals to corticosteroid treatment might be associated with poor prognosis [12, 13]. A prospective open-label trial (PRAISE study) is ongoing to compare the efficacy of steroids and supportive care in AIN [18]. In our cohort, 93.5% of patients were treated with immunosuppressives, mainly corticosteroids, immediately after biopsy-proven AIN diagnosis (two patients were not treated with immunosuppressives due to the improvement of renal functions after the cessation of the accused drugs for AIN). No statistical differences were observed between the efficacy of pulse therapy and oral steroids in our responder and non-responder patients. Despite the high rates of immunosuppressant use, renal recovery rates were not encouraging in our cohort, similar to previous studies [12, 13]. It could be related to chronic irreversible damages, such as tubular atrophy, interstitial fibrosis, and global glomerulosclerosis in significant number of patients. This was possibly related to the delayed diagnosis of AIN due to its asymptomatic clinical presentation. Typical clinical findings of AIN, such as fever, eosinophilia, and rash, which were described in early publications, are rare in AIN, according to previous studies [1, 2]. Although no statistically significant difference was present in terms of time from first symptom to biopsy between the responder and non-responder groups in our study, this interval was longer in the non-responder group. Besides the paucity of typical clinical findings of AIN, late admission or late referral of patients to our center could be another reason. Our hospital is a tertiary healthcare center, and most of the patients in our study group were referred from various hospitals. Also, the interval between first symptom to onset of immunosuppressive treatment was statistically longer in non-responders than responders. Hence, delayed diagnosis and late initiation of immunosuppressants might cause poor renal prognosis despite the high rates of immunosuppressive treatment in our cohort.

The study has limitations, including its retrospective nature, heterogeneous causes of AIN, limited number of patients, and single-center study design. In addition, COVID-19 or COVID-19 vaccine-related, IgG 4-related disease-associated, and immune checkpoint inhibitor-related AIN cases were not present among our cohort. Another limitation is that the etiology of AIN cannot be fully determined in some patients. Owing to the retrospective structure of our study, data were extracted from patient files, and thorough history, such as exposure to herbal remedies, could not be evaluated. On the other hand, long-term follow-up and outcome data, detailed treatment modalities, and laboratory and histopathological findings were the major strengths of our study. To the best of our knowledge, this is the first study to investigate the role of inflammatory indices, including NLR, PLR, and SII in AIN. In our cohort, the baseline PLR was lower in patients who progressed to ESKD, compared to the other markers, regardless of baseline serum creatinine and eGFR levels; however, due to the limited number of patients, these findings should be interpreted with caution.

In conclusion, AIN is a rare heterogeneous disease. Therefore, most studies have been retrospective case series. Compared to the biopsy series spread over a long-term period [13, 16, 19–21], we evaluated 31 cases elaborately in terms of clinical, histopathological, and laboratory findings, as well as treatment modalities, in this single-center study in a relatively short period (2006–2021); however, treatment responses were low in AIN, and 16.1% of patients progressed to ESKD despite high rates of immunosuppressive therapy, possibly due to delayed diagnosis and late initiation of immunosuppressants. We think our findings could provide new insights into the management of AIN patients. Nevertheless, studies including larger cohorts should be conducted to investigate the predictors of renal recovery and the optimal treatment for AIN.

## Supplementary Information

Supplemental Table 1: Clinical/laboratory and treatment data of ESKD and non-ESKD groups

Supplemental Table 2: Histopathological data of ESKD and non-ESKD groups

## References

[1] Sanchez-Alamo B, Cases-Corona C, Fernandez-Juarez G. Facing the challenge of drug-induced acute interstitial. Nephritis Nephron. 2023;147(2):78–90. 10.1159/000525561. 35830831 10.1159/000525561

[2] Naik RH, Annamaraju P. Interstitial Nephritis. In: StatPearls. Treasure Island: StatPearls; 2023. 2023 Jan. 33232019

[3] Szajek K, Kajdi ME, Luyckx VA, Fehr TH, Gaspert A, Cusini A, Hohloch K, Grosse P. Granulomatous interstitial nephritis in a patient with SARS-CoV‑2 infection. BMC Nephrol. 2021; 10.1186/s12882-020-02213-w. 33419393 10.1186/s12882-020-02213-w PMC7792557

[4] Donati A, Krishnan N. Should corticosteroids be used to treat biopsy-proven drug-induced acute interstitial nephritis?: PRO. Kidney. 2022;3(8):1306–9. 10.34067/KID.0006642021. 10.34067/KID.0006642021 PMC9416830 36176666

[5] de Hond TAP, Ocak G, Groeneweg L, Oosterheert JJ, Haitjema S, Khairoun M, Kaasjager KAH. Hematological ratios are associated with acute kidney injury and mortality in patients that present with suspected infection at the emergency department. J Clin Med. 2022;11(4):1017–1016. 10.3390/jcm11041017. 35207289 10.3390/jcm11041017 PMC8874958

[6] Willis K, Cheung M, Slifer SS, KDIGO board members. KDIGO 2012 clinical practice guideline for the evaluation and management of chronic kidney disease. Official J Int Soc Nephrol. 2012;3:1–163.

[7] Osumi T, Awazu M, Fujimura E, Yamazaki F, Hashiguchi A, Shimada H. Leukemia kidney infiltration can cause secondary polycythemia by activating hypoxia-inducible factor (HIF) pathway. Eur J Pediatr. 2013;172(6):829–32. 10.1007/s00431-013-2030-7. 23677251 10.1007/s00431-013-2030-7

[8] Bhat RA, Khan I, Khan I, Mir MA. Polycythemia, increased erythropoietin levels in a patient with renal lymphoma. Adv Biomed Res. 2014;26(3):147. 10.4103/2277-9175.135417. 10.4103/2277-9175.135417 PMC4139984 25161994

[9] Parlar H, Şaşkın H. Are pre and postoperative platelet to lymphocyte ratio and neutrophil to lymphocyte ratio associated with early postoperative AKI following CABG? Braz J Cardiovasc Surg. 2018;33(3):233–41. 10.21470/1678-9741-2017-0164. 30043915 10.21470/1678-9741-2017-0164 PMC6089132

[10] Zheng CF, Liu WY, Zeng FF, Zheng MH, Shi HY, Zhou Y, Pan JY. Prognostic value of platelet-to-lymphocyte ratios among critically ill patients with acute kidney injury. Crit Care. 2017; 10.1186/s13054-017-1821-z. 28882170 10.1186/s13054-017-1821-z PMC5590135

[11] Moledina DG, Wilson FP, Kukova L, Obeid W, Luciano R, Kuperman M, Moeckel GW, Kashgarian M, Perazella MA, Cantley LG, Parikh CR. Urine interleukin‑9 and tumor necrosis factor‑α for prognosis of human acute interstitial nephritis. Nephrol Dial Transplant. 2021;36(10):1851–8. 10.1093/ndt/gfaa169. 33125471 10.1093/ndt/gfaa169 PMC8476079

[12] Huang L, Liang S, Dong J, Fan W, Zeng C, Zhang T, Cheng S, Ge Y. Prognosis of severe drug-induced acute interstitial nephritis requiring renal replacement therapy. Ren Fail. 2021;43(1):1020–7. 10.1080/0886022X.2021.1942914. 34187299 10.1080/0886022X.2021.1942914 PMC8253213

[13] Hadded S, Harzallah A, Chargui S, Hajji M, Kaaroud H, Goucha R, Ben Hamida F, Gorsane I, Ben Abdallah T. Étiologies et facteurs pronostiques des néphropathies interstitielles aiguës. Nephrol Ther. 2021;17(2):114–9. 10.1016/j.nephro.2020.10.012. Etiologies and prognostic factors of acute interstitial nephritis. 33485789 10.1016/j.nephro.2020.10.012

[14] Moledina DG, Obeid W, Smith RN, Rosales I, Sise ME, Moeckel G, Kashgarian M, Kuperman M, Campbell KN, Lefferts S, Meliambro K, Bitzer M, Perazella MA, Luciano RL, Pober JS, Cantley LG, Colvin RB, Wilson FP, Parikh CR. Identification and validation of urinary CXCL9 as a biomarker for diagnosis of acute interstitial nephritis. J Clin Invest. 2023;133(13):e168950. 10.1172/JCI168950. 37395276 10.1172/JCI168950 PMC10313360

[15] Moledina DG, Wilson FP, Pober JS, Perazella MA, Singh N, Luciano RL, Obeid W, Lin H, Kuperman M, Moeckel GW, Kashgarian M, Cantley LG, Parikh CR. Urine TNF‑α and IL‑9 for clinical diagnosis of acute interstitial nephritis. JCI Insight. 2019;4(10):e127456. 10.1172/jci.insight.127456. 31092735 10.1172/jci.insight.127456 PMC6542610

[16] Clarkson MR, Giblin L, O’Connell FP, O’Kelly P, Walshe JJ, Conlon P, O’Meara Y, Dormon A, Campbell E, Donohoe J. Acute interstitial nephritis: clinical features and response to corticosteroid therapy. Nephrol Dial Transplant. 2004;19(11):2778–83. 10.1093/ndt/gfh485. 15340098 10.1093/ndt/gfh485

[17] Surendra M, Raju S, Chandragiri S, Uppin MS, Raju N. Steroid therapy in drug induced acute interstitial nephritis—Retrospective analysis of 83 cases. Saudi J Kidney Dis Transpl. 2019;30(1):157–65. 30804277

[18] Mose FH, Birn H, Hoffmann-Petersen N, Bech JN. Prednisolone treatment in acute interstitial nephritis (PRAISE)—protocol for the randomized controlled trial. BMC Nephrol. 2021; 10.1186/s12882-021-02372-4. 33933012 10.1186/s12882-021-02372-4 PMC8088674

[19] Muriithi AK, Leung N, Valeri AM, Cornell LD, Sethi S, Fidler ME, Nasr SH. Clinical characteristics, causes and outcomes of acute interstitial nephritis in the elderly. Kidney Int. 2015;87(2):458–64. 10.1038/ki.2014.294. 25185078 10.1038/ki.2014.294

[20] Muriithi AK, Leung N, Valeri AM, Cornell LD, Sethi S, Fidler ME, Nasr SH. Biopsy-proven acute interstitial nephritis, 1993–2011: a case series. Am J Kidney Dis. 2014;64(4):558–66. 10.1053/j.ajkd.2014.04.027. 24927897 10.1053/j.ajkd.2014.04.027

[21] Schwarz A, Krause PH, Kunzendorf U, Keller F, Distler A. The outcome of acute interstitial nephritis: risk factors for the transition from acute to chronic interstitial nephritis. Clin Nephrol. 2000;54(3):179–90. 11020015

