# Supplementary material for: Baseline systemic inflammatory indices and clinicopathological features to predict the outcome of acute tubulointerstitial nephritis: A single-center retrospective study
Source: Wien Klin Wochenschr. 2024 Aug 27;137(1-2):31–40. doi: 10.1007/s00508-024-02417-2 (PMC11739233; doi:10.1007/s00508-024-02417-2)
Supplement: Supplementary file 1 — Supplemental Table 1: Clinical/laboratory and treatment data of ESKD and non-ESKD groups [file 508_2024_2417_MOESM1_ESM.docx]

**Supplemental Table 1: Clinical/laboratory and treatment data of ESKD and non-ESKD groups**

| **Baseline (at time of renal bx) Parameters and follow-up data** | **ESKD patients (n=5)** | **Non-ESKD patients (n=26)** | **p value** |
| --- | --- | --- | --- |
| Age (Median) | 29 (21-55) | 49 (17-71) | 0.874 ^m^ |
| Gender, female/male | 3/2 | 22/4 | 0. 241 _F_ |
| Prior history of hypertension, n (%) | 1 (20) | 4 (15.4) | 1.000 _F_ |
| Prior history of diabetes mellitus, n (%) | 1 (20) | 1 (3.8) | 0.301 _F_ |
| Family history of kidney disease, n (%) | 0 (0) | 1 (3.8) | 1.000 _F_ |
| Time from first symptom to biopsy, median (days) (min-max) | 30 (5-61) | 16.5 (3-90) | 0.467 ^m^ |
| Serum albumin, median (min-max) | 4.18 (2.3-4.8) | 4.1 (2.7-4.74) | 0.845 ^m^ |
| Serum CRP, median (min-max) | 15.65 (1-87) | 34 (1-220) | 0.729 ^m^ |
| ESR, median (min-max) | 80 (33-101) | 69 (5-151) | 0.541 ^m^ |
| Serum LDH, median (min-max) | 217 (141-512) | 167 (129-335) | 0.657 ^m^ |
| Serum C3, median (min-max) | 118.5 (105-160) | 130 (73-201) | 0.954 ^m^ |
| Serum C4, median (min-max) | 33 (24-59) | 31 (20-79) | 0.974 ^m^ |
| **Hemoglobin,** median (min-max) | **12.05 (11.6-12.8)** | **9.9 (6.9-13.8)** | **0.033 ^m^** |
| **Lymphocyte,** median (min-max) | **2300 (1170-3300)** | **1260 (400-2800)** | **0.044 ^m^** |
| WBC, median (min-max) | 11250 (6030-20800) | 7680 (3900-29300) | 0.242 ^m^ |
| Neutrophil, median (min-max) | 7950 (4430-16100) | 5700 (3100-24700) | 0.333 ^m^ |
| Platelet, median (min-max) | 369000 (178500-494700) | 264000 (75000-645000) | 0.306 ^m^ |
| Eosinophil, median (min-max) | 200 (140-300) | 170 (0-700) | 0.346 ^m^ |
| SII, median (min-max) | 1441.83 (675.86-2000.3) | 1628 (495.7-5689.8) | 0.816 ^m^ |
| **PLR, median (min-max)** | **154.4 (124.2-197.9)** | **230.4 (151.6-1123.5)** | **0.016 ^m^** |
| NLR, median (min-max) | 3.82 (3-4.9) | 5.78 (2.29-17.6) | 0.188 ^m^ |
| Proteinuria (g/g or g/day), median (min-max) | 1.4 (0.1-8) | 0.83 (0-3.3) | 0.259 ^m^ |
| Time from first symptom to immunosuppressive treatment (days) (min-max) | 45 (13-76) | 27 (3-96) | 0.340 ^m^ |
| Mortality rate, n (%) | 1 (20) | 2 (7.7) | 0.422 _F_ |
| Treatments, n (%)  Steroids including pulse  AZA/MMF  Pulse steroids  Hemodialysis at admission | 5 (100)  2 (40)  1 (20)  1 (20) | 24 (92.3)  2 (7.7)  6 (23.1)  6 (23.1) | 1.000 _F_  0.112 _F_  1.000 _F_  1.000 _F_ |
| Etiology of AIN, n (%) | **Unknown, 5 (100)**  NSAID, (0)  Antibiotic, (0)  Sarcoidosis, (0)  Sjögren, (0) | **Unknown, 6 (23.1)**  NSAID, 7 (26.9)  Antibiotic, 7 (26.9)  Sarcoidosis, 4 (15.4)  Sjögren, 2 (7.7) | **0.003 _F_**  0.562 _F_  0.562 _F_  1.000 _F_  1.000 _F_ |

**Abbreviations:** AIN; Acute interstitial nephritis, AZA; Azathioprine, Bx; Biopsy, CRP; C reactive protein, eGFR; estimated glomerular filtration rate, ESR; Erythrocyte sedimentation rate, F: Fisher’s exact test, LDH; Lactate dehydrogenase, m; Mann-Whitney U, MMF; Mycophenolate mofetil, NLR; Neutrophil-to-lymphocyte ratio, NSAID; Non-steroidal anti-inflammatory drugs, PLR; Platelet-to-lymphocyte ratio, SII; Systemic immune inflammation index, WBC; White blood cell count

**Bold characters indicate statistically significant values**
