# Supplementary material for: Baseline systemic inflammatory indices and clinicopathological features to predict the outcome of acute tubulointerstitial nephritis: A single-center retrospective study
Source: Wien Klin Wochenschr. 2024 Aug 27;137(1-2):31–40. doi: 10.1007/s00508-024-02417-2 (PMC11739233; doi:10.1007/s00508-024-02417-2)
Supplement: Supplementary file 2 — Supplemental Table 2: Histopathological data of ESKD and non-ESKD groups [file 508_2024_2417_MOESM2_ESM.docx]

**Supplemental Table 2: Histopathological data of ESKD and non-ESKD groups**

| **Parameters** | **ESKD patients (n=5)** | **Non-ESKD patients (n=26)** | **p value** |
| --- | --- | --- | --- |
| Tubulitis, n (%)  Not significant  Mild  Moderate  Severe | 1 (20)  4 (80)  0 (0)  0 (0) | 4 (15.4)  19 (73.1)  1 (3.8)  2 (7.7) | 0.883 _X_^2^ |
| Lymphocytic infiltration, n (%)  Not significant  Mild  Moderate  Severe | 0 (0)  4 (80)  1 (20)  0 (0) | 1 (3.8)  19 (73.1)  4 (15.4)  2 (7.7) | 0.883 _X_^2^ |
| Eosinophilic infiltration, n (%)  Not significant  Mild  Moderate  Severe | 2 (40)  1 (20)  1 (20)  1 (20) | 15 (57.7)  7 (26.9)  1 (3.8)  3 (11.5) | 0.523 _X_^2^ |
| Plasmacytic infiltration, n (%)  Not significant  Mild  Moderate  Severe | 4 (80)  0 (0)  0 (0)  1 (20) | 18 (69.2)  1 (3.8)  1 (3.8)  6 (23.1) | 0.925 _X_^2^ |
| Granuloma formation, n (%)  No granuloma  Mild/microgranuloma  Moderate  Extensive | 3 (60)  2 (40)  0 (0)  0 (0) | 21 (80.8)  2 (7.7)  2 (7.7)  1 (3.8) | 0.240 _X_^2^ |
| **Tubular atrophy, n (%)**  None  Mild  Moderate  Severe | 1 (20)  3 (60)  1 (20)  0 (0) | 16 (61.5)  10 (38.5)  0 (0)  0 (0) | **0.030 _X_^2^** |
| **Interstitial Fibrosis, n (%)**  None  Mild  Moderate  Severe | 2 (40)  3 (60)  0 (0)  0 (0) | 22 (84.6)  3 (11.5)  1 (3.8)  0 (0) | **0.042 _X_^2^** |
| **Global sclerotic glomeruli (%), median (min-max)** | 41.9 (0-83.3) | 0 (0-60.6) | **0.014 ^m^** |
| Segmental sclerotic glomeruli (%), median (min-max) | 0 (0) | 0 (0-9) | 0.661 ^m^ |

**Abbreviations:** m: Mann-Whitney U, x^2^: Chi-square

**Bold characters indicate statistically significant values**
